# Lung-Targeted Delivery of Dimethyl Fumarate Promotes the Reversal of Age-Dependent Established Lung Fibrosis

**DOI:** 10.3390/antiox11030492

**Published:** 2022-02-28

**Authors:** Kosuke Kato, Ioannis Papageorgiou, Yoon-Joo Shin, Jennifer M. Kleinhenz, Sunny Palumbo, Seongmin Hahn, Joseph D. Irish, Skye P. Rounseville, Kenneth S. Knox, Louise Hecker

**Affiliations:** 1 Division of Pulmonary, Allergy and Critical Care and Sleep Medicine, Department of Medicine, Emory University, Atlanta, GA 30322, USA; kosuke.kato@emory.edu (K.K.); ioannis.papageorgiou@emory.edu (I.P.); yoon.joo.shin@emory.edu (Y.-J.S.); jennifer.m.kleinhenz@emory.edu (J.M.K.); 2 Division of Pulmonary, Allergy and Critical Care and Sleep Medicine, Department of Medicine, University of Arizona, Tucson, AZ 85721, USA; sunl@arizona.edu (S.P.); shahn@arizona.edu (S.H.); jirish12@arizona.edu (J.D.I.); srounseville@arizona.edu (S.P.R.); 3 Division of Pulmonary, Allergy and Critical Care and Sleep Medicine, Department of Medicine, University of Arizona College of Medicine-Phoenix, Phoenix, AZ 85004, USA; kknox@deptofmed.arizona.edu; 4 Atlanta VA Healthcare System, Atlanta, GA 30033, USA

**Keywords:** idiopathic pulmonary fibrosis, dimethyl fumarate, Nrf2, aging, antioxidant, reactive oxygen species

## Abstract

Idiopathic pulmonary fibrosis (IPF), a severe and deadly form of lung fibrosis, is widely regarded as a disease of aging. We previously demonstrated that aged mice with persistent lung fibrosis and IPF lung myofibroblasts exhibit deficient Nrf2-mediated antioxidant responses. Tecfidera is an orally administered FDA-approved drug for the treatment of multiple sclerosis, where the active pharmaceutical ingredient is dimethyl fumarate (DMF), an active Nrf2 activator. However, no studies have evaluated the efficacy of DMF for age-associated persistent lung fibrosis. Here, we demonstrate that in IPF lung fibroblasts, DMF treatment inhibited both TGF-β-mediated pro-fibrotic phenotypes and led to a reversal of established pro-fibrotic phenotypes. We also evaluated the pre-clinical efficacy of lung-targeted (inhaled) vs. systemic (oral) delivery of DMF in an aging murine model of bleomycin-induced persistent lung fibrosis. DMF or vehicle was administered daily to aged mice by oral gavage or intranasal delivery from 3–6 weeks post-injury when mice exhibited non-resolving lung fibrosis. In contrast to systemic (oral) delivery, only lung-targeted (inhaled) delivery of DMF restored lung Nrf2 expression levels, reduced lung oxidative stress, and promoted the resolution of age-dependent established fibrosis. This is the first study to demonstrate the efficacy of lung-targeted DMF delivery to promote the resolution of age-dependent established lung fibrosis.

## 1. Introduction

Human fibrotic disorders affect many organ systems, including the heart, blood vessels, kidneys, liver, and lungs [1,2]. An estimated 45% of deaths in the U.S. are attributable to disorders that are characterized by varying degrees of fibrosis [2]. The most severe form of lung fibrosis is idiopathic pulmonary fibrosis (IPF), a fatal and relentlessly progressive disorder [3,4]. IPF is characterized by excessive scar tissue formation and irreversible destruction of the lung parenchyma, resulting in gas-exchange abnormalities and ultimately respiratory failure. Although two drugs have gained FDA approval for IPF (nintedanib and pirfenidone), neither are shown to definitively improve quality of life or survival. Further, although both drugs are shown to inhibit the development of fibrosis, we recently reported that nintedanib failed to demonstrate efficacy for reversing age-associated established lung fibrosis [5]. Clearly, improved therapies for the treatment of IPF and other fibrotic diseases are needed. 

As the average life expectancy continues to increase, the elderly population is growing at a rapid pace. Progressive fibrosis is a hallmark of aging in various organ systems, including the liver [6], kidneys [7], pancreas [8], and lungs [9]. With this shift in the elderly demographic, it has become increasingly important to understand the contribution of aging to disease pathogenesis. IPF is widely regarded as a disease of aging [10,11,12]. The incidence and prevalence of IPF increase with age; two-thirds of IPF patients are older than 60 years at the time of presentation, with a mean age of 66 years at the time of diagnosis [4]. Further, the survival rate for IPF patients markedly decreases with age [10]. Despite this strong association, cellular/molecular mechanisms that account for the aging predilection to fibrotic disease are not well understood. 

Key mechanisms of pathological fibrosis appear to be common among all tissues/organs, in particular oxidative stress and myofibroblast activation. Oxidative stress is defined as an imbalance between reactive oxygen species (ROS) production and antioxidant capacity. Numerous studies implicated defective antioxidant responses in the pathogenesis of IPF [13,14,15,16,17,18]. The induction of the nuclear factor erythroid 2–related factor 2 (Nrf2) serves as a master regulator of the cellular antioxidant responses by inducing the transcription of a wide array of genes that can mitigate oxidative stress. However, patients with IPF exhibit decreased Nrf2 expression and redox imbalance that promotes pro-fibrogenic responses in myofibroblasts [19,20,21]. In animal models of pulmonary fibrosis, Nrf2 deficiency results in heightened oxidative stress and more severe pulmonary fibrosis [22,23,24]. Our previous studies demonstrated that aged mice exhibited an impaired capacity for fibrosis resolution; this is in part regulated by alterations in cellular redox homeostasis resulting from deficient induction of Nrf2 in lung fibroblasts [19]. Although the efficacy of antioxidant-targeted strategies via Nrf2 activation is shown to suppress the development of bleomycin-induced lung fibrosis in young animals [25,26], the efficacy of Nrf2 activation for reversing age-dependent established lung fibrosis has never been evaluated. 

Although therapeutic strategies targeting Nrf2 for the treatment of IPF hold promise, systemic delivery of antioxidant-targeted strategies (such as N-Acetylcysteine) failed in clinical trials for IPF (NCT00639496) [27]. Further, systemic targeting of Nrf2 has a high potential for off-target effects as well as safety concerns; systemic delivery of the Nrf2 activator, bardoxolone methyl, was terminated in phase III clinical trials due to safety concerns (NCT01351675). Tecfidera (developed by Biogen) is an FDA-approved drug for the treatment of multiple sclerosis via the oral route of administration; the active pharmaceutical ingredient in Tecfidera is dimethyl fumarate (DMF), an Nrf2-activator [28]. There is a critical need for antioxidant-based therapies that can promote the resolution of established lung fibrosis without deleterious adverse effects. The goal of this study was to determine the pre-clinical efficacy of lung-targeted delivery vs. systemic delivery of DMF in an aging murine model of persistent lung fibrosis. 

## 2. Materials and Methods

### 2.1. Reagents

We purchased the following reagents: Dimethyl sulfoxide (DMSO) from Mediatech (Tewksbury, MA, USA); Hank’s Balanced Salt Solution (HBSS, -Ca/Mg), Dulbecco’s Modified Eagle Medium (DMEM), and DMF from Fisher Scientific (Waltham, MA, USA); Penicillin/streptomycin solution and fetal bovine serum (FBS) from Gibco (Grand Island, NY, USA); Amplex Red reagent from Molecular Probes (Eugene, Oregon); CellTiter-Glo^®^ Luminescent reagent from Promega (Madison, WI, USA); human recombinant TGF-β1 from R&D Systems (Minneapolis, MN, USA). We purchased the following antibodies: anti-collagen-1α from Invitrogen (Carlsbad, CA, USA); anti-fibronectin-1, anti-Nrf2, and anti-β-actin from Abcam (Cambridge, MA, USA); anti-GAPDH from Cell Signaling (Danvers, MA, USA); horseradish peroxide-conjugate secondary antibodies from Bio-Rad (Hercules, CA, USA). All other chemicals and reagents were purchased from Sigma-Aldrich unless otherwise specified.

### 2.2. Cell Culture

Fibroblasts were isolated from the lung of a patient with IPF using collagenase digestion as previously described [19] under protocols approved by the institutional review boards of the University of Arizona (IRB protocol #1200000347). All cells were cultured in DMEM supplemented with 10% FBS, 100 U/mL penicillin, 100 μg/mL streptomycin, and 1.25 μg/mL amphotericin B, at 37 °C in 5% CO_2_, 95% air [19].

### 2.3. Amplex Red Assay

Hydrogen peroxide (H_2_O_2_) levels in blood and H_2_O_2_ generated by fibroblasts were determined by the Amplex Red assay kit (Life Technologies, Eugene, OR) as previously described [29]. The fluorescence intensity was measured at 525 nm for excitation and emission in the range of 580–640 nm using the Glomax multi detection system (Promega, Madison, WI, USA). 

### 2.4. Western Blotting

We prepared cell lysates and lung homogenates in RIPA buffer, subjected them to SDS-PAGE under reducing conditions, and performed Western blotting as previously described [30]. Lysates were quantitated using a Micro BCA Protein assay kit (Pierce, Waltham, MA, USA) according to the manufacturer’s instructions. We used enhanced chemiluminescence Western blotting substrate and c400 Imager (Azure Biosystems, Dublin, CA, USA) to detect specific immunoreactive signals. Densitometric analysis was performed using the NIH ImageJ software. 

### 2.5. Murine Model of Bleomycin-Induced Lung Injury

Aged (18 months) female C57BL/6J mice (The Jackson Laboratory) were anesthetized using intraperitoneal injection of ketamine (100 mg/kg) and xylazine (10 mg/kg). Bleomycin (0.02875 U/mouse; 50 μL) was administered intratracheally to induce lung fibrosis as previously described [19]. Since gender differences exist in response to injury in this model [31], we utilized female mice only to avoid sex-related variability of responses to injury. 

### 2.6. Therapeutic Treatments

At 3 weeks post-bleomycin administration, mice were randomly assigned to one of four treatment groups. Between 3–6 weeks post-injury, DMF or vehicle (sterile PBS) was administered daily by oral gavage (240 μg/dose in 150 µL total volume) (Table 1) using a gavage tube. In parallel, mice were anesthetized using isoflurane and administered DMF or vehicle (sterile PBS) daily by intranasal instillation (80 μg/dose in 50 µL total volume) (Table 1). The control animals received vehicle only. Dosing was based on the solubility of this compound in the vehicle in the total volume used for administration. All mice were monitored for body-weight changes and survival. Mice were removed from the study when we observed greater than 20% weight loss compared to pre-surgery (day 0) or lack of responsiveness to touch. At 6 weeks post-injury, all mice were sacrificed by CO_2_ inhalation. All procedures were approved by the Institutional Animal Care and Use Committee (IACUC, #14-535 approved on 17 July 2017) at the University of Arizona.

### 2.7. Oxidized Glutathione Assay

Oxidized glutathione levels in the lung homogenates were determined by the glutathione assay kit (Cayman Chemicals, Ann Arbor, MI, USA) according to the manufacturer’s instructions. Oxidized glutathione was normalized to the total protein content in the lung homogenate.

### 2.8. Histology

The lungs were fixed overnight in 10% neutral buffered formalin, processed, embedded in paraffin, and the sections were stained with hematoxylin and eosin (H&E) or Masson’s trichrome staining was performed [30].

### 2.9. Hydroxyproline Assay

The lungs were weighed, homogenized in sterile water, and hydrolyzed in 12N HCl at 120 °C for 3 h. A hydroxyproline assay (Sigma-Aldrich, St. Louis, MO, USA) was performed according to the manufacturer’s instructions using hydroxyproline as a standard. Hydroxyproline concentration was normalized to the total protein content in the lung homogenate.

### 2.10. Lipid Peroxidation Assay

Oxidized lipid levels in the lung homogenates were determined by the Lipid peroxidation assay kit (Cayman Chemicals, Ann Arbor, MI, USA) according to the manufacturer’s instructions. Oxidized lipid concentration was normalized to the total protein content in the lung homogenate.

### 2.11. Statistical Analysis

Graphs were generated, and statistical analyses were performed with GraphPad Prism (GraphPad Software, San Diego, CA, USA). Data are expressed as means ± SEM. Differences among groups were assessed with one-way ANOVA multiple comparisons with a Tukey’s post-test and between pairs with Student’s two-tailed *t*-test. *p* < 0.05 is considered statistically significant (* *p* < 0.05, ** *p* < 0.01, and *** *p* < 0.001).

## 3. Results

### 3.1. DMF Treatment Reduces ROS Levels in IPF Lung Fibroblasts

**Figure 1:**
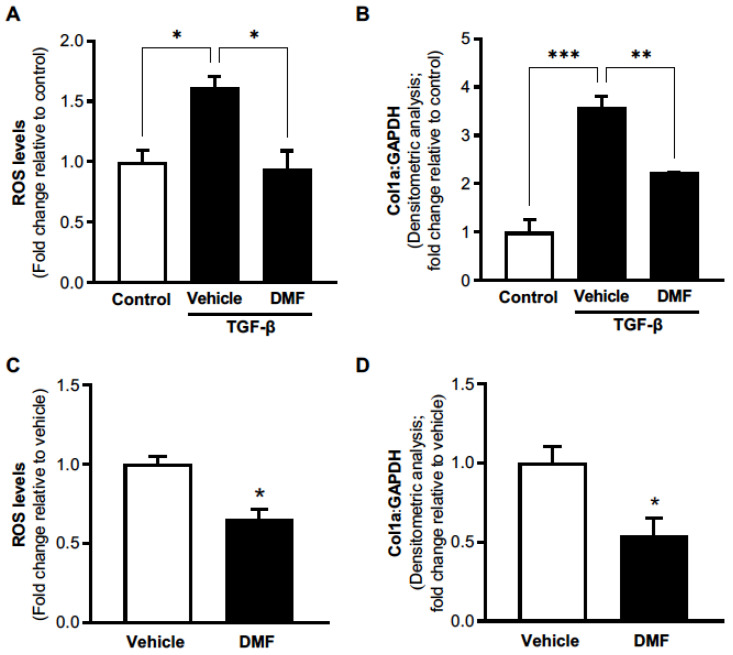
DMF treatment inhibits pro-fibrotic phenotypes in IPF lung fibroblasts. Primary fibroblasts were isolated from the lung of a patient with biopsy-proven IPF. (**A**,**B**) IPF lung fibroblasts were treated with DMF (1 µM) or vehicle (DMSO) followed by treatment ± TGF-β (2 ng/mL). H_2_O_2_ levels were evaluated at 24 h by Amplex Red assay (**A**), and whole-cell lysates were assessed for collagen-1α expression at 48 h by Western blot; densitometric analyses are shown (**B**). (**C**,**D**) IPF lung fibroblasts were treated with DMF (1 µM) or vehicle (DMSO). Culture supernatant was evaluated for H_2_O_2_ levels at 24 h by Amplex Red assay (**C**), and whole-cell lysates were assessed for collagen-1α expression at 24 h by Western blot; densitometric analyses are shown (**D**). All values represent means  ±  SEM; technical replicates (*n* = 3); * *p* < 0.05; ** *p* < 0.01; *** *p* < 0.01 using Student’s two-tailed *t*-test to compare the means between two groups and one-way ANOVA multiple comparisons with a Tukey’s post-test to compare the means among three groups.

Numerous studies implicated defective antioxidant responses in the pathogenesis of IPF [13,14,15,16,17,18]. In IPF lung fibroblasts, decreased Nrf2 expression was associated with pro-fibrogenic phenotypes [20]. Further, Nrf2 activation (via sulforaphane treatment) restored antioxidant responses and promoted dedifferentiation of IPF myofibroblast [20]. In line with these findings, our previous studies demonstrated that IPF lung myofibroblasts exhibit defective Nrf2 responses associated with oxidative stress [19]. DMF treatment was shown to potently diminish pro-fibrotic phenotypes in lung fibroblasts isolated from patients with systemic sclerosis [25]. However, the efficacy of DMF was not previously evaluated in IPF lung fibroblasts. Since primary mesenchymal cells isolated from IPF lungs represent a heterogeneous population of both fibroblasts and myofibroblasts “(myo)fibroblasts”, we sought to evaluate the efficacy of DMF on both TGF-β-induced and established pro-fibrogenic phenotypes of these cells. To determine if DMF treatment can inhibit fibrogenic responses induced by TGF-β, cells were pre-treated with DMF or vehicle followed by treatment with TGF-β for 24 h. DMF treatment significantly reduced TGF-β-induced production of ROS, specifically H_2_O_2_, as compared to vehicle treatment (Figure 1A). DMF treatment also led to the inhibition of TGF-β-induced expression of collagen-1α, a major ECM component in IPF fibroblasts (Figure 1B, Supplemental Figure S1). Since primary IPF lung (myo)fibroblasts exhibit enhanced production of ROS and ECM [32], we next sought to determine if DMF can reverse these established pro-fibrotic phenotypes. DMF treatment significantly inhibited the production of H_2_O_2_ (Figure 1C) and collagen-1α (Figure 1D, Supplemental Figure S1), as compared to vehicle treatment. These results support the concept that activation of Nfr2 by DMF could be a therapeutic approach to target redox imbalance and fibrogenic responses in IPF.

### 3.2. Oral DMF Delivery Fails to Reverse Age-Dependent Persistent Lung Fibrosis

**Figure 2:**
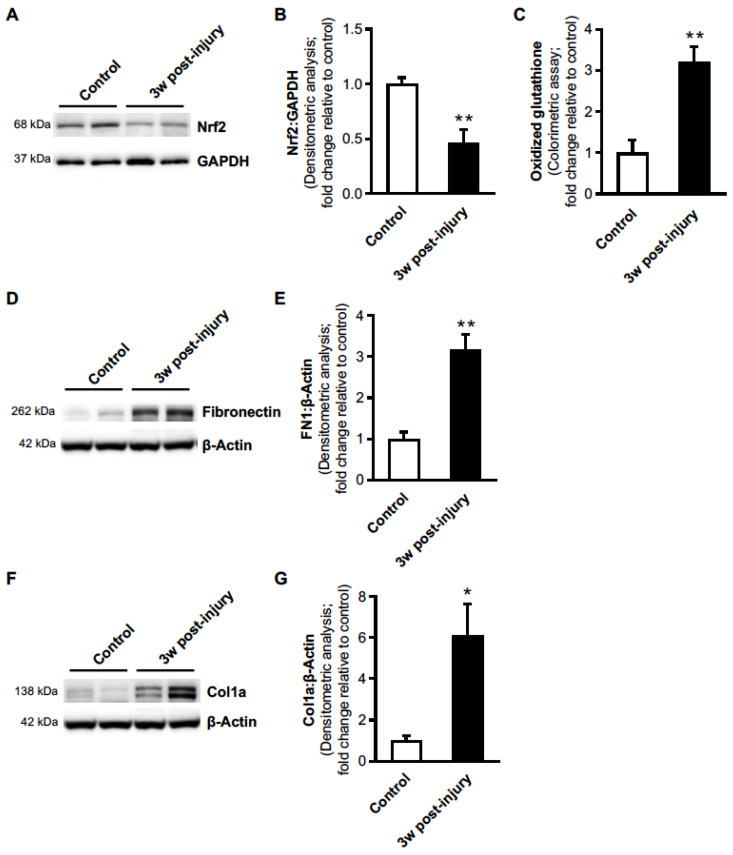
Aged-injured mice exhibit Nrf2 deficiency associated with oxidative stress and lung fibrosis. (**A**,**B**) Lung protein was assessed for Nrf2 expression by Western blot (**A**) and densitometric analysis (**B**). (**C**) The level of lung oxidized glutathione was analyzed by quantitative oxidized glutathione assay. (**D**–**G**) Lung protein was assessed for fibronectin (**D**,**E**) and collagen-1α (Col-1α) (**F**,**G**) expression by Western blot and densitometric analysis. Representative Western blot images are shown. All values represent means of biological replicates ± SEM; *n* = 4 mice per group; * *p* < 0.05; ** *p* < 0.01 using Student’s two-tailed *t*-test.

**Figure 3:**
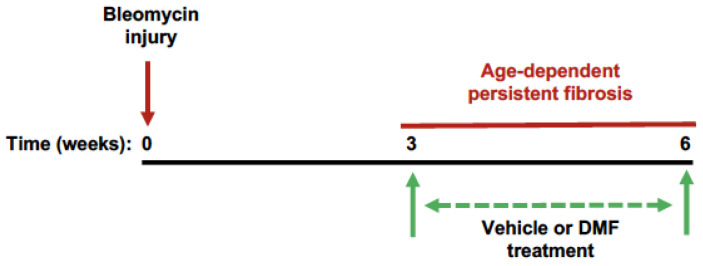
Schematic diagram illustrating age-dependent persistent lung fibrosis and treatment protocol. C57BL/6J aged (18 months) female mice received an intratracheal instillation of bleomycin (0.02875 U/mouse). DMF was administered daily by oral gavage (240 µg/150 µL in sterile PBS/mouse) or intranasal instillation (80 µg/50 µL in sterile PBS/mouse) from 3–6 weeks post-injury.

**Figure 4:**
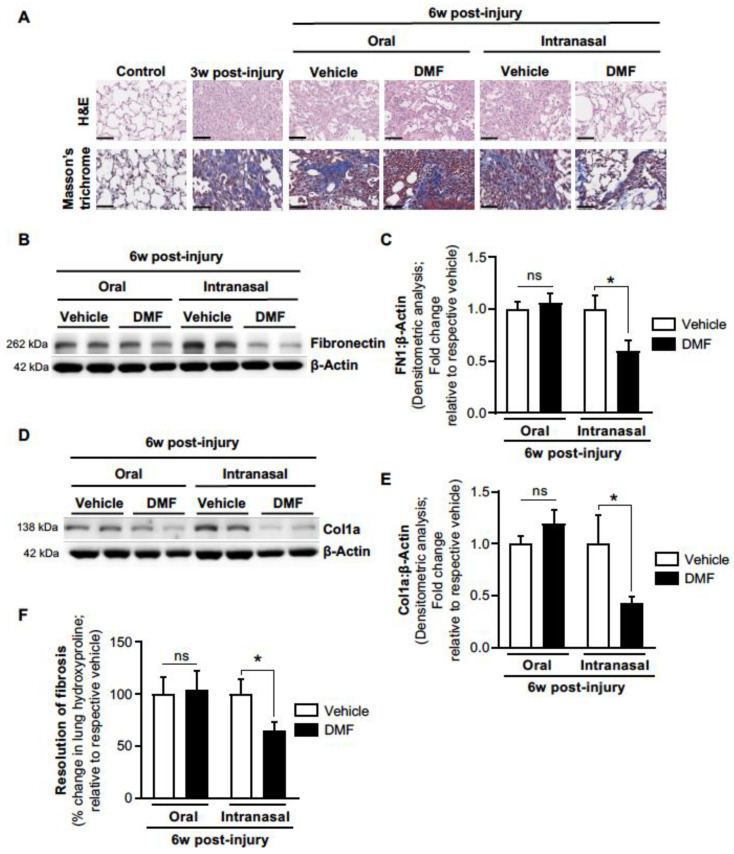
Intranasal (but not oral gavage) administration of DMF promotes reversal of age-associated established lung fibrosis. (**A**) Lung tissue was assessed by H&E staining for histopathology (top panels) and Masson’s trichrome blue staining for collagen (bottom panels). (**B**–**E**) Lung protein extract was assessed for fibronectin (**B**,**C**) and collagen-1α (**D**,**E**) expression by Western blot and densitometric analysis. Representative Western blot images are shown. Oral-vehicle (*n* = 6); oral-DMF (*n* = 5); intranasal-vehicle (*n* = 4); intranasal-DMF (*n* = 6). (**F**) The resolution of fibrosis was analyzed by quantitative hydroxyproline assay. Oral-vehicle (*n* = 5); oral-DMF (*n* = 5); intranasal-vehicle (*n* = 5); intranasal-DMF (*n* = 6). All values represent means of biological replicates ± SEM; ns: not significant; * *p* < 0.05 using Student’s two-tailed *t*-test.

In young mice, bleomycin-induced injury results in a self-limited fibrotic response, where fibrosis spontaneously resolves following peak injury [19,33]. Conversely, aged mice exhibit a persistent fibrotic response, with little to no resolution of fibrosis from 3 weeks to 4 months post-injury [19]. We previously demonstrated that Nrf2 levels are highly upregulated in young mice following bleomycin-induced injury, which promotes fibrosis resolution [19]. In contrast, the lack of fibrosis resolution in aged mice is associated with a deficient Nrf2 response, where Nrf2 levels are significantly downregulated [19]. Although a recent study demonstrated that daily intraperitoneal administration of DMF inhibited the development of bleomycin-induced lung fibrosis in young mice [25], no studies evaluated whether DMF can reverse age-dependent established lung fibrosis (which is associated with deficient Nrf2 levels). We first sought to confirm that aged mice exhibit a deficient Nrf2 response following bleomycin-induced lung injury. As expected, aged mice demonstrated a significant decrease in lung Nrf2 expression levels at 3 weeks post-injury (Figure 2A,B), which was accompanied by increased levels of oxidized glutathione (Figure 2C), and increased expression of ECM markers, including fibronectin (Figure 2D,E) and collagen-1α (Figure 2F,G). This aging model of bleomycin-induced persistent lung fibrosis enables the evaluation of therapeutic agents for their efficacy in reversing age-dependent established fibrosis [5]. Therefore, we sought to determine whether systemic delivery of DMF (via oral gavage) demonstrates efficacy for reversing age-dependent established fibrosis. Starting at 3 weeks post-injury (when aged mice exhibit established/persistent fibrosis) [19], DMF or vehicle was administered by oral gavage daily through 6 weeks (Figure 3). Similar to our previously reported findings [19], aged vehicle-treated mice demonstrated persistent lung fibrosis at 6 weeks post-injury, as demonstrated by histopathology (Figure 4A; top panels), lung collagen deposition (Figure 4A; bottom panels), elevated fibronectin (Figure 4B,C) and collagen-1α (Figure 4D,E) expression levels, and elevated lung hydroxyproline levels (Figure 4F). However, oral DMF treatment failed to promote the resolution of age-dependent established lung fibrosis during this 3-week treatment period, as lung fibrosis in mice treated with oral DMF was indistinguishable from vehicle-treated mice (Figure 4A–F). Overall, systemic (oral) administration of DMF failed to reverse age-dependent established lung fibrosis. 

### 3.3. Intranasal DMF Delivery Promotes Resolution of Age-Dependent Established Fibrosis

Inhaled drug delivery offers advantages over systemic (oral) drug administration for lung-targeted indications, as higher drug concentrations can be delivered directly to the lungs while potentially reducing the concentration of systemic exposure among other organs. Therefore, we sought to evaluate the efficacy of lung-targeted DMF treatment (via inhaled delivery) for reversing age-dependent established fibrosis (as opposed to systemic delivery, which failed to demonstrate efficacy). Using the same injury model and treatment protocol, aged mice were administered DMF or vehicle daily via intranasal delivery from 3–6 weeks post-injury (Figure 3). Inhaled DMF treatment resulted in resolution of age-dependent established lung fibrosis, as demonstrated by histopathology (Figure 4A; top panels), reduced collagen deposition (Figure 4A; bottom panels), significantly decreased expression levels of fibronectin (Figure 4B,C) and collagen-1α (Figure 4D,E), and decreased lung hydroxyproline levels (Figure 4F) as compared to vehicle-treated and oral DMF treated mice (Figure 4A–F). In summary, these data suggest that, although systemic DMF delivery failed to reverse established fibrosis, lung-targeted DMF treatment via inhaled delivery demonstrated efficacy for promoting the resolution of age-dependent established lung fibrosis.

### 3.4. Intranasal DMF Delivery Rescues Deficient Nrf2-Mediated Antioxidant Responses and Promotes Lung Redox Homeostasis

**Figure 5:**
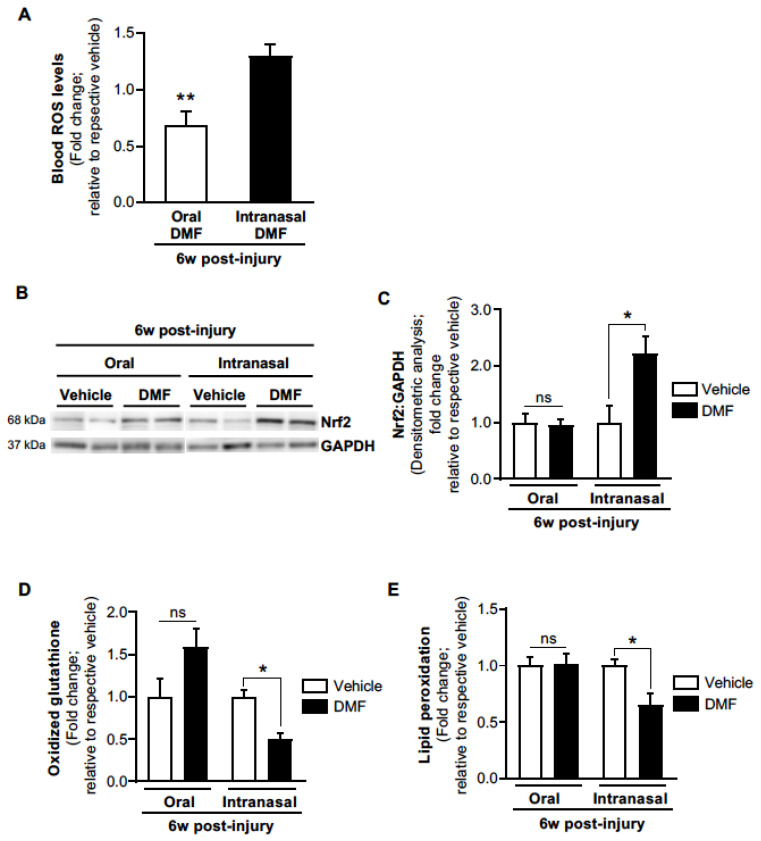
Intranasal (but not oral gavage) administration of DMF restores lung Nrf2 levels in aged-injured mice. (**A**) Blood ROS levels were analyzed by Amplex Red assay. Oral-DMF (*n* = 7); intranasal-DMF (*n* = 6). (**B**,**C**) Lung protein extract was assessed for Nrf2 expression by Western blot (**B**) and densitometric analysis (**C**). Representative Western blot images are shown. Oral-vehicle (*n* = 7); oral-DMF (*n* = 7); intranasal-vehicle (*n* = 4); intranasal-DMF (*n* = 4). (**D**) The level of lung oxidized glutathione was analyzed by a quantitative oxidized glutathione assay. Oral-vehicle (*n* = 5); oral-DMF (*n* = 5); intranasal-vehicle (*n* = 3); intranasal-DMF (*n* = 4). (**E**) The level of lung lipid peroxidation was analyzed by quantitative lipid peroxidation assay. Oral-vehicle (*n* = 5); oral-DMF (*n* = 5); intranasal-vehicle (*n* = 4); intranasal-DMF (*n* = 3). All values represent means of biological replicates ± SEM; ns: not significant; * *p* < 0.05; ** *p* < 0.01 using Student’s two-tailed *t*-test.

Given that lung-targeted inhaled delivery of DMF demonstrated efficacy for promoting the resolution of age-dependent established lung fibrosis, whereas systemic oral delivery failed to demonstrate efficacy, we next evaluated the pharmacodynamics of oral vs. inhaled DMF delivery. Although oral administration of DMF led to reduced blood ROS levels (Figure 5A), it failed to rescue deficient Nrf2 expression levels in the lungs of aged mice with established fibrosis (Figure 5B,C). In contrast, although intranasal DMF delivery did not impact blood ROS levels (Figure 5A), it resulted in significantly increased lung Nrf2 expression levels (Figure 5B,C). Further, intranasal DMF delivery also led to significantly reduced oxidized glutathione levels (Figure 5D) and lipid peroxidation (Figure 5E) in the lungs, as compared to vehicle or oral DMF treatment. These data suggest that inhaled DMF delivery promotes Nrf2-mediated lung antioxidant responses to rescue lung redox imbalance without affecting systemic redox levels. In summary, lung-targeted treatment with DMF via inhaled delivery (as compared to oral delivery) can more effectively rescue local Nrf2 expression levels and promote redox homeostasis in the lungs. 

## 4. Discussion

Oxidative stress has long been associated with fibrotic disorders, including IPF [34]. Despite the clear link between aging and oxidative stress, therapeutically tenable agents that target age-associated oxidative stress to treat fibrosis have yet to be translated into a viable treatment for patients with IPF. Pre-clinical studies demonstrated a critical role for Nrf2 in mediating antioxidant responses during injury-repair. Further, Nrf2 activators, including sulforaphane [26] and DMF [25], demonstrated efficacy for inhibiting the development of fibrosis in young mice. However, in aged mice, lung injury results in persistent fibrosis, which is associated with defective Nrf2-mediated antioxidant responses [19]. Further, Nrf2 expression was reportedly decreased in the lungs of patients diagnosed with IPF [20]. Although therapeutic strategies targeting Nrf2 for the treatment of IPF hold promise, Nrf2-targeted strategies yet to be evaluated in clinical trials for IPF. Systemic delivery of the Nrf2 activator, Bardoxolone methyl, was terminated in phase III clinical trials for pulmonary hypertension due to safety concerns, suggesting that long-term systemic treatment with some Nrf2 activators may not be appropriate for chronic diseases such as IPF. Tecfidera (an Nrf2 activator via the active pharmaceutical ingredient, DMF) is an FDA-approved drug for multiple sclerosis via oral administration. However, DMF was not evaluated for its efficacy in reversing age-associated established fibrosis. We hypothesized that strategies that more directly target the source(s) of redox imbalance via localized tissue-specific delivery would offer greater potential to reduce unintended side-effects and would be more effective as compared to systemic delivery for reversing age-associated established fibrosis. Our studies indicate that while systemic (oral) DMF delivery attenuated blood ROS levels, it failed to rescue lung Nrf2 levels and did not demonstrate efficacy for resolving age-dependent established lung fibrosis. Importantly, lung-targeted DMF delivery rescued deficient Nrf2-mediated antioxidant responses within the lung and promoted the resolution of age-dependent established lung fibrosis. The current study provides a side-by-side comparison of systemic vs. local delivery of DMF and demonstrates proof-of-concept for a lung-targeted antioxidant approach as a therapeutic strategy for reversing age-associated established fibrosis.

A major limitation of the traditional bleomycin-induced lung fibrosis model in young mice is the resolving nature of fibrosis, as lung injury results in a limited fibrotic response which resolves beyond 3 weeks post-injury [19,33]. One potential explanation for the limited clinical translation of therapeutics for IPF is that age-dependent pathologic mechanisms remain largely unexploited in the drug development process, despite the fact that aging is strongly implicated in the pathogenesis of IPF [34,35]. Pre-clinical animal models of lung fibrosis are largely performed in young mice (8–10 weeks), which predominantly results in a self-limited fibrotic response [36]. Treatment interventions are largely preventative (dosing before or at the time of injury) rather than curative [37]. Previous pre-clinical studies demonstrating the efficacy of Nrf2 activators for inhibiting the development of lung fibrosis in young animals using preventative treatment schedules [25,26] are useful for initial proof-of-concept. However, it has become increasingly important to re-evaluate the use of this model for pre-clinical studies to predict therapeutic benefit in subsequent clinical trials, particularly since numerous drug candidates demonstrating efficacy in young mice have failed in clinical trials with elderly patients. This aging murine model of non-resolving lung fibrosis better recapitulates pathological signaling observed in human IPF patients (e.g., defective Nrf2 responsiveness), as limited knowledge of age-dependent pathological mechanisms/targets remain key challenges and may in part explain the limited therapies available. This is the first study that leverages an animal model of persistent lung fibrosis to evaluate the pre-clinical efficacy of an Nrf2 activator DMF for reversing established fibrosis (versus evaluating the pre-clinical efficacy of inhibiting de novo synthesis of fibrosis); this is a more clinically relevant efficacy testing protocol since patients with IPF typically present with well-established fibrosis. With the emergence of senolytics as a new potential strategy for IPF [38,39], age-relevant pre-clinical efficacy models have become increasingly important to enhance the potential for clinical translation. Gender differences exist in response to bleomycin-induced lung injury [31]; therefore, we used female mice only to avoid sex-related variability of responses to injury. Future studies could further validate these findings in male mice. Overall, this study sheds light on the utility of aging animal models, as they provide opportunities to perform more rigorous efficacy testing that may enable the selection of more effective drug candidates, such as Tecfidera, which can promote the resolution of age-dependent established fibrosis. Leveraging aging models at the appropriate stages of therapeutic development is likely to provide novel insight that would accelerate the successful translation of improved therapies for IPF.

The two currently FDA-approved drugs for IPF slow disease progression, although they are not shown to reverse fibrosis [5], and neither drug is shown to definitively improve quality of life or survival for patients. Further, a number of side effects are reported for these drugs, which are administered via the oral route. There are currently two blockbuster dry powder inhalers (DPI) used most in the U.S. and globally, Advair and Spiriva (both are prescribed for asthma and COPD indications). From the clinical standpoint, lung-targeted drug delivery offers several major advantages over systemic delivery routes, including the ability to deliver higher local drug concentration and the potential for reduced side effects. Our study indicates that lung-targeted delivery of DMF, but not systemic delivery, mediates upregulation of Nrf2 expression, antioxidant activity, and promotes partial resolution of age-dependent established lung fibrosis. Of note, although the oral dose was three times higher (12 mg/kg) than the inhaled dose (4 mg/kg), only inhaled delivery demonstrated efficacy for promoting reversal of established fibrosis, despite the lower dose. Thus, although current IPF therapies are administered orally, lung-targeted delivery of novel therapeutics may provide greater efficacy (even at lower doses with reduced side effects. To date, no therapeutics have been shown to reverse age-associated established fibrosis, which may represent the holy grail for therapeutic strategies to more effectively treat IPF. A better understanding of injury-repair in the context of aging and/or efficacy evaluation of drug candidates in age-relevant pre-clinical models are likely to more accurately predict clinical translation of therapies for IPF, a disease that disproportionately afflicts the elderly population.

## Figures and Tables

**Table 1:** DMF Formulation Protocol.

Delivery Route	Vehicle	Volume	Dose
Intranasal	PBS	50 μL	80 μg
Oral gavage	PBS	150 μL	240 μg

DMF was dissolved in sterile PBS at 42 °C and sonicated for 50 min.

## Data Availability

Data is contained within the article.

## Supplementary Materials

The following supporting information can be downloaded at: https://www.mdpi.com/article/10.3390/antiox11030492/s1.
