# Supplementary material for: Lung-Targeted Delivery of Dimethyl Fumarate Promotes the Reversal of Age-Dependent Established Lung Fibrosis
Source: Antioxidants (Basel). 2022 Feb 28;11(3):492. doi: 10.3390/antiox11030492 (PMC8944574; doi:10.3390/antiox11030492)
Supplement: Supplementary file 1 [file antioxidants-11-00492-s001.zip › antioxidants-1534495-supplementary.pdf]

Supplemental figure 1

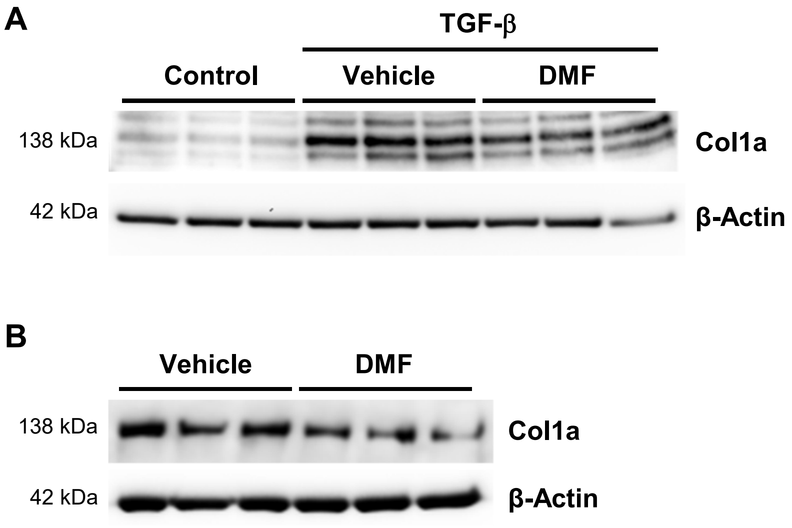

Supplemental Figure S1. DMF treatment inhibits pro-fibrotic phenotypes in IPF lung fibroblasts. (**A**) IPF lung fibroblasts were treated with DMF (1 μM) or vehicle (DMSO) followed by treatment +/- TGF-β (2 ng/mL). Whole-cell lysates were assessed for collagen-1α expression at 48h by Western blot. (**B**) IPF lung fibroblasts were treated with DMF (1 μM) or vehicle (DMSO). Whole-cell lysates were assessed for collagen-1α expression at 24h by Western blot.
